# A case of giant cellulitis-like Sweet syndrome and review

**DOI:** 10.1016/j.jdcr.2025.08.013

**Published:** 2025-08-22

**Authors:** Sarah A. Amjad, Thais Pincelli, Olayemi Sokumbi

**Affiliations:** a Mayo Clinic Alix School of Medicine, Jacksonville, Florida; b Department of Dermatology, Mayo Clinic, Jacksonville, Florida; c Department of Laboratory Medicine & Pathology, Mayo Clinic, Jacksonville, Florida

**Keywords:** cellulitis, giant cellulitis-like Sweet syndrome, neuroendocrine tumor, neutrophilic dermatoses, obesity, Sweet syndrome

## Introduction

Neutrophilic dermatoses such as Sweet syndrome (SS) are defined by the presence of a noninfectious, neutrophilic infiltrate in the dermis and/or hypodermis. SS, also known as acute febrile neutrophilic dermatosis, is characterized by an aseptic dermal neutrophilic infiltrate.^1^ It presents with sudden development of tender erythematous plaques associated with fever, leukocytosis, and general malaise.^2^^,^^3^ These isolated or recurrent episodes can affect any part of the body, most commonly the neck and extremities.^4^ There are several subtypes of SS, including classical, drug-induced, malignancy-associated, necrotizing, and a rare variant known as giant cellulitis-like Sweet syndrome (GCSS).^5^^,^^6^ GCSS was first described by Surovy et al in 2013 and is characterized by extensive, erythematous plaques involving the suprapubic region, buttocks, trunk, and extremities.^1^ Lesions are painful, warm, strongly infiltrated, and can exceed 50-60 cm in diameter. Plaques can exhibit vesicular or bullous changes with a purpuric component, while others are more discrete and mildly infiltrated.^1^ In contrast to other variants of SS, it is associated most frequently with obesity but has also been linked with autoimmune diseases and hematologic malignancies.^1^^,^^2^ Due to their similar clinical presentations, GCSS is commonly misdiagnosed as cellulitis. However, the lack of response to antibiotics helps exclude cellulitis.^7^ A skin biopsy can confirm the diagnosis of GCSS. We report a case of GCSS in an obese patient with a recent history of duodenal neuroendocrine tumor and end-stage renal disease (ESRD) along with a comprehensive review of previously published cases.

## Case summary

**Fig 1:**
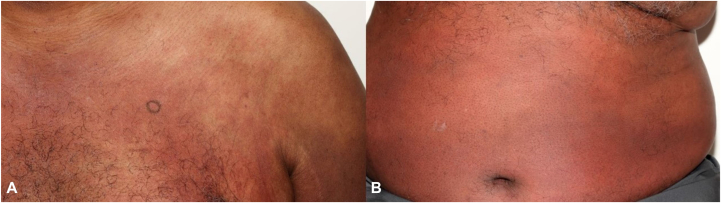
Extensive tender, warm, erythematous plaques on the upper chest **(A)** and abdomen **(B)**.

**Fig 2:**
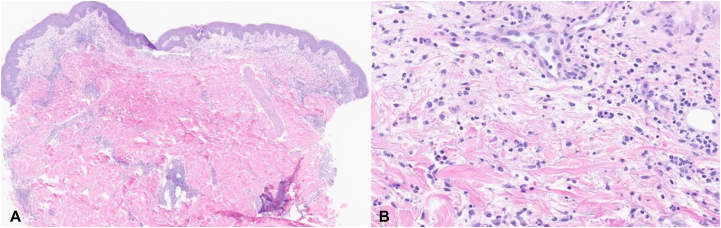
**A,** Papillary dermal edema with a superficial and deep perivascular and interstitial infiltrate (hematoxylin & eosin; ×5). **B,** The infiltrate is comprised of neutrophils, rare nuclear dust, and eosinophils (hematoxylin & eosin; ×40).

A 64-year-old obese man with a history of duodenal neuroendocrine tumor, ESRD on dialysis, and renal transplant failure on 10 mg prednisone daily presents with a 3-month history of a recurrent pruritic eruption involving his trunk and extremities, as well as low-grade fever. When present, this rash flares for 3 to 4 days, then disappears. Prior to presentation, the patient was evaluated at the emergency room for the rash and received a 7-day course of cephalexin and a taper dose of prednisone 0.4 mg/kg daily, with significant improvement. The patient denied any changes in medications, exposures, changes in household products, or recent travel since the rash developed. Regarding the duodenal tumor, about 4 months prior, a duodenal biopsy showed a well-differentiated neuroendocrine tumor. Subsequent Gallium-68 DOTA-Tyrosine3-Octreotate positron emission tomography–computed tomography was unremarkable; therefore, no additional treatment was needed at the time, with follow-up endoscopy recommended. Examination revealed extensive erythematous to violaceous plaques on the upper chest and abdomen, extending to the proximal upper and lower extremities, flanks, and lower back bilaterally (Fig 1). The eruption was warm to touch, in contrast to the surrounding areas of normal skin. Postinflammatory hyperpigmentation was noted on the upper back. Laboratory tests showed hemoglobin of 10.9 g/dL (normal 13.2-16.6 g/dL), normal leukocyte count (7300/μL; range 3400-9600/μL) with neutrophilia (84.9% neutrophils) and lymphopenia (8.2% lymphocytes), normal platelet levels, erythrocyte sedimentation rate of 53 mm/h (normal 0-22 mm/h), and C-reactive protein of 112 mg/L (normal <5.0 mg/L). All microorganism cultures of the skin tissue were negative. Other autoimmune and inflammatory tests were within normal limits. A biopsy showed papillary dermal edema and superficial and deep perivascular and interstitial infiltrate comprised predominantly of neutrophils, rare nuclear debris, and scattered eosinophils (Fig 2). There were no microorganisms identified with special stains. The patient was subsequently diagnosed with GCSS and started on 0.4 mg/kg/day prednisone for 10 days with complete resolution of the eruption. At 9 months following treatment, the patient had not reported any recurrent episodes of GCSS to the clinical team.

## Discussion

In our case of recurrent GCSS, there was a history of obesity, duodenal neuroendocrine tumor, and ESRD status post-renal transplant on dialysis. Previous reports of GCSS have also been associated with obesity,^1^^,^^2^^,^^4^^,^^7^, ^8^, ^9^ as well as hematologic^1^^,^^2^^,^^4^^,^^10^, ^11^, ^12^ and other solid organ malignancies (breast and gastric adenocarcinoma),^1^^,^^6^^,^^12^ and postsurgery^5^^,^^9^ (summarized in Table I).

**Table I:** Literature review of previous cases of GCSS

	Age/sex	Relevant history	Presentation	Labs	Location	Rash gross description	Histologic description	Duration of disease	Episodic or recurrent	Antibiotics given?	Treatment
Surovy et al, 2013^1^	62 M	Obesity, multiple myeloma	Fever and malaise	WBC of 10,600/μL, platelets 96,000,000/μL, CRP 279 mg/L	Left leg, buttocks, and trunk	Warm, erythematous plaques with vesicular, bullous, and purpuric components	Dermal edema with an inflammatory infiltrate of mature neutrophils with no evidence of leukocytoclastic vasculitis	2 y, 9 episodes, 4 wk each	Recurrent	Yes	Prednisone
Surovy et al, 2013^1^	48 F	Obesity	Fever and malaise	WBC of 24,000/μL, CRP 429 mg/L	Right lower leg and knee, left arm, buttocks, and trunk	Painful, warm erythematous plaques with a bullous and purpuric component	Dermal edema with an inflammatory infiltrate of mature neutrophils with no evidence of leukocytoclastic vasculitis	8 y, frequent episodes	Recurrent	No	Prednisone
Surovy et al, 2013^1^	68 F	Obesity, breast cancer	Fever and malaise	WBC of 11,300/μL, CRP 235 mg/L	Suprapubic region, right thigh, leg, and foot	Erythematous plaques with vesicular, bullous, and purpuric components	Dermal edema with an inflammatory infiltrate of mature neutrophils with no evidence of leukocytoclastic vasculitis	3 mo, 3 episodes	Recurrent	No	Topical corticosteroid
Kaminska et al, 2014^8^	54 F	Sjögren's disease, obesity, PBC with liver transplant	Fever and chills	WBC of 4100/μL (77% neutrophils)	Buttocks, extremities, trunk, head, and neck.	Erythematous, edematous, indurated plaques	Papillary dermal edema with perivascular interstitial neutrophils, lymphocytes, histiocytes, and eosinophils in the absence of leukocytoclastic vasculitis	2 d	Episodic	Yes	Prednisone
Koketsu et al, 2014^7^	60s F	Obesity	Fever	WBC of 10,300/μL, neutrophils, 8755/μL	Right thigh and flank	Large erythematous plaque	Papillary dermal edema with perivascular interstitial neutrophils and lymphocytes in the absence of leukocytoclastic vasculitis	3 y, frequent episodes	Recurrent	Yes	Colchicine, prednisone, and dapsone
So et al, 2015^10^	72 F	Myelodysplastic/proliferative syndrome	Fever	WBC of 73.5/uL (neutrophils 66%), absolute neutrophil count of 58.1/uL, hemoglobin of 10.6 g/dL	Left leg and flank	Sharply demarcated erythematous plaque containing petechiae	Papillary dermal edema with perivascular interstitial histiocytoid, immature granulocytic cells, lymphocytes, eosinophils, neutrophils, extravasated erythrocytes, hemosiderin-laden macrophages, and focal areas of vessel damage.	2 wk	Episodic	Yes	Clobetasol
Okuyama et al, 2019^12^	56 F	Gastric cancer, trauma to leg, AML	Fever	(multiple values in Fig 1 of original publication)	Bilateral legs	Cellulitis-like eruption	Dermal and subcutaneous adipose infiltrate composed of neutrophils with no evidence of leukocytoclastic vasculitis	205 d, frequent episodes	Recurrent	Yes	Prednisolone, indomethacin, and surgical debridement
Mitaka et al, 2020^6^	90 F	Breast cancer	Confusion and generalized weakness	WBC of 29,100/μL (78% neutrophils)	Right breast, axilla, flank, and lower extremity	Pruritic, well-demarcated, erythematous blanching patches	Dense dermal neutrophilic infiltrate with no evidence of leukocytoclastic vasculitis	2 wk	Episodic	Yes	Prednisone
Zhao et al, 2022^11^	52 M	Myelodysplastic syndrome, sternal aspiration	Fever and chills	WBC of 1820/μL, absolute neutrophil count of 1070/μL, lymphocyte count of 600/μL, platelets of 19,000/μL, and hemoglobin of 5.3 g/dL	Sternal aspiration site	Painful, violaceous, and swollen nodule with surrounding erythema	Papillary dermal edema with perivascular interstitial myeloperoxidase + CD163-mononuclear cells without vasculitis	1 wk	Episodic	Yes	Prednisone
Hirt et al, 2023^13^	70 F	SARS-CoV-2 booster vaccine, Sweet syndrome	Fever	None	Gluteal area to thigh	Large erythematous, indurated plaque	Dermal edema with dense neutrophilic infiltrate	7 y prior, recurred after vaccine, treated within 1 wk	Recurrent	Yes	Intramuscular triamcinolone
Bae et al, 2023^5^	69 F	Varicose vein surgery s/p 1 week	Fever	88.8% neutrophils, CRP 91.4 mg/L, ESR 43 mm/h.	Right thigh	Painful, erythematous plaques with vesicular and bullous components	Dermal edema and diffuse neutrophils with histiocytoid mononuclear cells without vasculitis	1 wk	Episodic	Yes	Dexamethasone
Li et al, 2023^2^	62 M	Obesity, CML	Fever, malaise, cough, congestion, and arthralgias	91% neutrophils, CRP 246 mg/L	Back, buttocks, and thighs	Nonblanching, erythematous plaque with vesicular and bullous components	Dermal neutrophilic infiltration with no evidence of leukocytoclastic vasculitis	1 wk	Episodic	Yes	Prednisone
Hingtgen et al, 2023^4^	36 M	Obesity, CML, CHF	Fever, chills, diaphoresis, and nausea	WBC of 172/μL (64% neutrophils and 34% bands)	Axilla, flank, and chest	Painful erythematous plaque	Dermal edema with perivascular interstitial neutrophils	1 wk	Episodic	Yes	Prednisone
Díez-Madueño et al, 2024^9^	53 M	Obesity, endoscopic ureteral lithotripsy s/p 1 mo	Fever and malaise	WBC of 11,980/μL (80% neutrophils), absolute neutrophil count of 9600/μl, CRP of 198 mg/l	Left thigh, flank, buttock, and lumbosacral region	Large, well-defined erythematous-edematous plaque	Papillary dermal edema with perivascular interstitial lymphocytes and neutrophils without leukocytoclastic vasculitis	6 y, frequent episodes	Recurrent	Yes	Corticosteroid, colchicine, and dapsone
Muche et al, 2024^3^	60 M	None given	Fever, chills, sweating, upper extremity/chest pain, and rigor	WBC of 17,590/μL (88.4% neutrophils; 3.6% lymphocytes), platelets 88,000/μL, CRP 200 mg/L	Chest, right neck, and medial arm	Painful erythematous plaques with ill-defined borders	Papillary dermal edema with perivascular interstitial neutrophils, lymphocytes, and rare eosinophils associated with dermatitis in the absence of vasculitis	3 wk	Episodic	Yes	Prednisone
Our case	64 M	Obesity, ESRD, kidney transplant	None	WBC of 7300/μL (84.9% neutrophils; 8.2% lymphocytes), ESR 53 mm/hr, CRP 112.0 mg/L	Upper arms, abdomen, flanks, and lower back	Warm, erythematous plaques with hyperpigmentation	Papillary dermal edema with perivascular interstitial neutrophils with rare eosinophils.	3 mo, frequent episodes that last 3-4 d	Recurrent	Yes	Prednisone

*CHF*, Congestive heart failure; *CML*, chronic myeloid leukemia; *CRP*, C-reactive protein; *ESR*, erythrocyte sedimentation rate; *ESRD*, end-stage renal disease; *F*, female; *GCSS*, giant cellulitis-like Sweet syndrome; *M*, male; *WBC*, white blood cell.

Although the association of GCSS with obesity is well-recognized, to our knowledge, there has never been a case reported with duodenal neuroendocrine tumor or renal transplant specifically. There has been one previous case of GCSS associated with liver transplant, but the patient had concurrent Sjögren disease, which may explain the development of GCSS.^8^ Like prior reports of chronic relapsing GCSS, the biopsy demonstrated strong dermal edema with only mild neutrophilic infiltration.^1^ In contrast to episodic GCSS with more prominent neutrophilic infiltrate. Notably, there are a few reports of histiocytoid GCSS, both characterized by histiocyte infiltration and GCSS-like cutaneous lesions associated with myelodysplastic or proliferative syndromes.^10^^,^^11^

The clinical differential diagnosis for GCSS includes cellulitis, erysipelas, Well's cellulitis, erysipeloid plaques of congenital autoinflammatory diseases, reactive angioendotheliomatosis, the localized relapsing form of neutrophilic dermatosis, and other neutrophilic dermatoses. All subtypes of SS, including GCSS, are driven by activation and infiltration of neutrophils into the dermis in the absence of infection and suggest complex mechanisms of autoinflammation.^1^^,^^4^ Therefore, laboratory findings in GCSS typically demonstrate neutrophilic leukocytosis along with elevated inflammatory markers, including erythrocyte sedimentation rate and C-reactive protein, and potentially mild anemia and thrombocytosis.^1^^,^^2^^,^^9^ In malignancy-associated cases, leukopenia or thrombocytopenia can be observed.^4^^,^^12^ Clinically, GCSS is similar to cellulitis and erysipelas, presenting with tenderness, swelling, and erythema of the affected skin, fever, and leukocytosis. However, on histopathology, GCSS displays abundant dermal neutrophilic inflammatory infiltration with no evidence of infectious organisms or vascular dilation typical of erysipelas. In addition to fever, GCSS can present with other systemic symptoms like malaise and arthralgias. Similar to GCSS, Well’s cellulitis and localized relapsing neutrophilic dermatoses have both been associated with a variety of systemic symptoms. However, localized relapsing neutrophilic dermatoses typically occur in areas of tissue damage or injury.^14^, ^15^, ^16^ Rather than dermal neutrophilic inflammation, Well’s cellulitis is characterized by eosinophilic infiltrates and flame figures, and reactive angioendotheliomatosis features vascular proliferation and thrombi.^16^^,^^17^ Finally, erysipeloid plaques in congenital autoinflammatory disease typically present at a younger age, and there is typically a suggestive family history.^16^ Overall, a thorough history of potential predisposing factors and frequency of episodes, appropriate clinicopathological correlation, and response to anti-inflammatory treatment allow for distinguishing GCSS from its various mimickers.

Although no clinical trials have been conducted, the most common treatment for GCSS is oral prednisone. Other therapies utilized in the documented cases include dapsone,^7^^,^^9^ intramuscular dexamethasone,^5^ topical clobetasol,^10^ indomethacin,^12^ and intramuscular triamcinolone.^13^ Surgical exploration was done in 1 case, with no evidence of necrotizing skin infection or abscess.^4^ Surgical debridement was employed in 1 hospitalized patient with extensive eruption on the lower extremities after trying three different antibiotics with no improvement.^13^

GCSS is associated with autoimmune disease and malignancy, while many of its mimickers are not, so proper medical history and screening for malignancy should be considered if GCSS is suspected or diagnosed. Given that GCSS can resemble other more common conditions, clinicopathologic correlation is essential for an accurate diagnosis. This ensures appropriate and timely treatment, leading to the resolution of symptoms.

## Conflicts of interest

None disclosed.
